# c-Src and EGFR Inhibition in Molecular Cancer Therapy: What Else Can We Improve?

**DOI:** 10.3390/cancers12061489

**Published:** 2020-06-07

**Authors:** Stefania Belli, Daniela Esposito, Alberto Servetto, Ada Pesapane, Luigi Formisano, Roberto Bianco

**Affiliations:** Department of Clinical Medicine and Surgery, University of Naples “Federico II”, 80131 Naples, Italy; stefania.bell21@gmail.com (S.B.); daniela.esposito1989@gmail.com (D.E.); Alberto.Servetto@UTSouthwestern.edu (A.S.); ada.pesapane@libero.it (A.P.)

**Keywords:** Src kinase family, c-Src inhibitors, EGFR, EGFR-TKIs, drug resistance

## Abstract

The proto-oncogene c-Src is a non-receptor tyrosine kinase playing a key role in many cellular pathways, including cell survival, migration and proliferation. c-Src de-regulation has been observed in several cancer types, making it an appealing target for drug discovery efforts. Recent evidence emphasizes its crucial role not only in promoting oncogenic traits, but also in the acquisition and maintenance of cancer resistance to various chemotherapeutic or molecular target drugs. c-Src modulates epidermal growth factor receptor (EGFR) activation and amplifies its downstream oncogenic signals. In this review, we report several studies supporting c-Src kinase role in the intricate mechanisms of resistance to EGFR tyrosine kinase inhibitors (TKIs). We further highlighted pre- and clinical progresses of combined treatment strategies made in recent years. Several pre-clinical data have encouraged the use of c-Src inhibitors in combination with EGFR inhibitors. However, clinical trials provided controversial outcomes in some cancer types. Despite c-Src inhibitors showed good tolerability in cancer patients, no incontrovertible and consistent clinical responses were recorded, supporting the idea that a better selection of patients is needed to improve clinical outcome. Currently, the identification of biological markers predictive of therapy response and the accurate molecular screening of cancer patients aimed to gain most clinical benefits become decisive and mandatory.

## 1. Introduction

SRC is a representative member of nine-gene family of non-receptor tyrosine kinases (Src Family Kinases, SFKs) playing a key role in the modulation of several signaling pathways. As a cytoplasmic protein c-Src regulates cellular responses to external stimuli through interaction with multiple proteins [1]. Focal-adhesion proteins, adaptor proteins and transcription factors are included in its complex network of interactions, which support c-Src role in the direct and indirect modulation of mitogenic signaling, cytoskeletal organization, angiogenesis, motility, cell cycle progression, survival and proliferation [2,3].

Structurally, c-Src consists of seven functional domains: 1) an N-terminal myristoylation sequence attached to a Src homology 4 (SH4) domain required for cellular membrane localization; 2) a unique domain, which provides unique functions and specificity to each SFK member, followed by 3) SH3 and 4) SH2 domains, important for protein–protein interaction and for the binding of phosphorylated tyrosine sites, respectively; 5) a linker region, involved in intramolecular binding to the SH3 domain; 6) a protein tyrosine-kinase region, also known as SH1 domain, representing the catalytic domain bearing the auto-phosphorylation site Tyrosine (Y) 419 and 7) a short C-terminal regulatory segment carrying an auto-inhibitory phosphorylation site, the Y530 [1,4,5]. Conformational changes in the molecular structure determine the activation and status of the c-Src protein. The phosphorylation of the C-terminal Y530 blocks the protein in a closed, inactive conformation, which masks the kinase domain, making it inaccessible to substrate proteins. This inhibitory phosphorylation at c-Src C-terminal region is fine-tuned by c-Terminal Src kinase (CSK). Conversely, c-Src activation occurs with the de-phosphorylation of the C-terminal site (i.e., by the protein tyrosine phosphatase 1B, PTP1B), which dissociates it from the SH2 domain, inducing c-Src in an open, active state. However, to fully obtain c-Src activation the Y419 auto-phosphorylation is required [6,7,8]. c-Src activation can be promoted also by CRK-associated substrate (CAS) and focal adhesion kinase (FAK) bindings to the c-Src SH2 and SH3 domains, leading in turn to the disruption of the inhibitory intramolecular interactions and allowing c-Src activation [9,10]. Likewise, activated growth-factor receptors can associate with the c-Src SH2 domain, prompting c-Src activation by a similar mechanism [1,8,11]. The intricate regulation of this pleiotropic protein increases the risk to alter c-Src levels and activity, events extensively studied in cancer. Although a truncated c-Src C-terminal region that exhibits constitutive catalytic activity was detected in small subsets of colon and endometrial cancers [12,13], the genetic mutations of c-Src represent a rare event in cancer development and progression. More commonly, increased expression and/or activity of wild type c-Src protein have been described in a number of human cancers, including lung, skin, colon, pancreatic, prostate, breast, ovarian, endometrial, and head and neck malignancies [14,15]. The effects of c-Src alteration in cancer tissues vary from motility and invasion to proliferation, apoptosis and angiogenesis [14,16,17], playing a critical role in the development of malignant phenotype. The c-Src activity can be modulated by protein kinases/phosphatases (i.e., the above mentioned CSK and PTP1B) regulating c-Src switch from inactive to active state, or can be boosted by alterations of its upstream or downstream partners. c-Src, indeed, interacts with several protein-tyrosine kinase receptors at the plasma membrane, producing a bi-directional flow of information: receptors affect c-Src activity and vice versa. Several studies defined the increased activity of c-Src as a result of the altered interactions with ligand-activated receptor tyrosine kinases, such as epidermal growth factor receptor (EGFR) [18,19,20], platelet derived growth factor receptor (PDGFR) [21], fibroblast growth factor receptor (FGFR14–) [22], colony stimulating factor-1 receptor (CSF-1R) [23,24], human epidermal growth factor 2 (HER2/neu) [19] and hepatocyte growth factor receptor (c-Met) [25].

Here, we describe the physical and functional interaction between c-Src and EGFR—both ubiquitously expressed and often over-expressed and/or mutated (i.e., EGFR) in cancer cells—and their involvement in cancer as well as in drug resistance. Particularly, we elucidate the role of c-Src in resistance to EGFR inhibitors and the current pre-clinical and clinical progresses of combined therapy of c-Src and EGFR inhibitors.

## 2. c-Src and EGFR Physical and Functional Interaction

c-Src and EGFR have been shown to enhance pro-mitogenic signals upon epidermal growth factor (EGF) stimuli [26]. c-Src and activated EGFR cooperate to induce cell transformation and cancer development [27]. c-Src has been reported to bind to EGFR and phosphorylate tyrosine residues on its C-terminal domain, resulting in a variety of downstream effects. Particularly, c-Src-mediated EGFR activation involves tyrosine residues different from the auto-phosphorylation sites, including Y891, Y920, Y1101 and, most notably, Y845 [28,29,30,31]. Y845 is located within the catalytic domain of EGFR in a conserved position among all receptors and non-receptor tyrosine kinases that generally undergoes auto-phosphorylation to induce receptor catalytic activity. Nevertheless, Y845 phosphorylation of EGFR is mediated by c-Src and not by EGFR itself. Sato and colleagues hypothesized that EGF binding to EGFR triggers a conformational change in its kinase domain that allows Y845 to be accessible for c-Src recruitment, hence providing a docking site for physical interaction with either SH2 domain of c-Src itself and with other signaling molecules [32].

c-Src activation induced by EGFR ligands mediates the binding of phosphatidylinositol 3-kinase (PI3K) to EGFR, leading to AKT phosphorylation and, in turn, induction of survival and migration signaling pathways [30,33,34,35,36,37]. Additionally, although c-Src needs the contribution of other molecules to modulate the proliferative mitogen-activated protein kinase (MAPK) pathway [18], it has been demonstrated that it can enhance EGFR ligands-induced extracellular signal-regulated protein kinase 1/2 (ERK1/2) activation, in particular through phospholipase C γ-1 (PLCγ-1) or Raf-1 [38,39]. The significant role of c-Src in MAPK activation was further demonstrated using Src family inhibitor PP2, which partially prevented EGF-induced ERK1/2 activation [40].

These data suggested that pY845 requires several mediators for accomplishing the synergism between EGFR and c-Src, including for instance signal transducer and activator of transcription 5B (STAT5b), a transcription factor involved in mitogenesis [41], and cytochrome c oxidase subunit II (Cox II) [42], a mitochondrially encoded protein involved in oxidative phosphorylation and in cytochrome c release during apoptosis. In particular, pY845 is involved in both STAT5b regulation, responsible of EGF-induced cell proliferation and DNA synthesis [41,43], and in enhancing cell survival through Cox II.

c-Src is also engaged in EGFR activation by responding to extracellular stimuli other than EGFR ligands. c-Src, indeed, is required for EGFR trans-activation induced by multiple extracellular factors, such as G protein-coupled receptor ligands, steroids, cytokines, extracellular matrix proteins, ionizing radiation, ultraviolet light, and certain ions [44,45,46]. Moro and colleagues, for instance, elucidated the c-Src requirement for EGFR trans-activation following its association with integrins [47]. c-Src mediates the cross-talk between EGFR and other non-related membrane receptors and regulates the relative downstream effects through Y845 phosphorylation. It has been demonstrated that c-Src induces EGFR phosphorylation following G protein-coupled receptors (GPCR) activation. Src-specific inhibitors or the expression of mutated EGFR-Y845 reduced lysophosphatidic acid (LPA)-induced DNA synthesis [14,45], indicating that c-Src-induced phosphorylation of Y845 is crucial for the mitogenic response to both EGFR and GPCR (the LPA-receptor) [18,45]. c-Src modulation is also involved in the trans-activation of EGFR by endothelin receptor [48], transforming growth factor receptor (TGFR) [49], phorbol myristate acetate (PMA) receptor [50], Insulin receptor [51] and b2 adrenergic G-protein coupled receptor [52]. Finally, c-Src exhibited a key role in the internalization and degradation of EGFR. To promote internalization, c-Src modulates phosphorylation of clathrin and dynamin, involved in the formation of the coated pits embracing ligand-bound receptors and in the separation of the endocytic vesicles from the plasma membrane, respectively [53,54]. EGFR degradation, instead, is triggered by E3 ubiquitin-protein ligase Cbl (Cbl) ubiquitination, which promotes receptor endocytosis and degradation [55]. c-Src affects this process by promoting the ubiquitination and proteasomal degradation of Cbl, which in turn postpones EGFR degradation and down-regulation, thus inducing EGFR recycling to the plasma membrane and the recovery of its signaling [53].

## 3. c-Src and EGFR Activation and Cooperation in Cancer Onset and Maintenance

After the discovery of c-Src and EGFR cooperation in cellular processes, many researchers focused on the understanding of how this interaction and its downstream signaling can deregulate cellular functions, pushing malignant cell transformation. Since the first studies, it has been suggested that the synergism between c-Src and EGFR contributes to a more aggressive phenotype in diverse tumors. Maa and collaborators verified that concomitant over-expression of c-Src and EGFR in murine fibroblasts led to a higher tumorigenic phenotype compared to cells over-expressing either the EGFR and c-Src alone [27,56]. Both c-Src and EGFR have been found co-overexpressed in several types of tumor, including glioblastomas and carcinomas of the colon, breast, and lung [31,57,58,59]. In lung cancer, c-Src over-expression is observed in 50–80% of non-small cell lung cancer (NSCLC) patients and is related to poor clinical outcome, which has increased the interest in using c-Src kinase inhibitors as therapeutic cancer agents [60,61]. EGFR over-expression and mutations, as well, play a key role in the carcinogenesis of NSCLC and frequently occur. Interestingly, Sonnweber and colleagues have shown that in a cohort of stage I NSCLC patients, the phosphorylation of Y845 on EGFR was a valuable prognostic factor—more than the incidence of the EGFRvIII mutation [62]. Lin and colleagues demonstrated that digoxin, a cardiac glycoside suggested as chemo-therapeutic agent, induced decrease of c-Src, EGFR and STAT3 activation and expression and, consequently, impaired cancer cell proliferation, migration and invasion [63]. Additionally, Lai and colleagues identified rhodomycin A as a promising compound for inhibiting c-Src activity in NSCLC. It also led to the decrease of Src-associated proteins, including EGFR, STAT3, and FAK. Interestingly, the inhibition of Src-related signaling pathways—such as PI3K, c-Jun N-terminal kinases (JNK), Paxillin, and p130cas—significantly inhibited in vitro and in vivo tumorigenicity of NSCLC cells [64], confirming the crucial role of c-Src and EGFR in these tumors. Finally, focusing on the role of tumor microenvironment in c-Src/EGFR regulation, Interleukin 10 (IL10) has been proposed as a cooperative agent in the oncogenic progression of lung cancer by increasing phosphorylation levels of EGFR and c-Src in a dose-dependent manner. IL10 induced Janus chinasi 1 (JAK1)/STAT3 activation through the recruitment of pSrc to pIL10 receptor, resulting in the up-regulation of EGFR expression. The latter event led to an increased transcription and mRNA stability of IL10 by EGFR itself, producing a positive feedback for EGFR over-expression that triggered lung cancer tumorigenesis [65].

In breast cancer, c-Src is over-expressed in ~70% of cases and, in the majority of them, is co-overexpressed with at least one member of EGFR family [14], suggesting their cooperation in promoting breast cancer development. Dimri and colleagues demonstrated that the concomitant over-expression of both EGFR and c-Src, but not of EGFR or c-Src alone, markedly cooperate to enhance breast cancer cells oncogenic properties, causing hyper-proliferation, aberrant three-dimensional acinar structures, increased migration and invasion, and anchorage-independent cell growth [66]. In 2011, Irwin and colleagues reported that EGFR and c-Src co-localized into lipid rafts in triple negative breast cancer cells, and that this co-localization prompted cell sensitivity to simultaneous treatment with EGFR and c-Src inhibitors. The authors described PI3K also associated with lipid rafts and that the inhibition of c-Src activity decreased AKT phosphorylation, suggesting c-Src regulation of PI3K/AKT survival signals within lipid rafts [67].

The involvement of c-Src in HER2-mediated cellular processes (such as anchorage-independent growth, motility, and survival) has been largely elucidated in breast cancer [68,69]. The over-expression of HER2 in mammary epithelial cells [70] or in HER2-expressing transgenic mouse systems [71] has been correlated with the activation of c-Src kinase, suggesting a functional interaction between c-Src and HER2 in breast cancer pathogenesis. Moreover, HER2 up-regulates c-Src protein levels by increasing its protein synthesis, activating the AKT/mTOR/4E-BP1 pathway, and stability inhibiting calpain-mediated Src protein degradation. The over-expression of c-Src, in turn, markedly enhances the ability of HER2 to promote invading and metastatic traits [69,70,72]. Successively, the pivotal role of c-Src upstream HER2 has been demonstrated by Ishizawar and colleagues, who found that the over-expression of c-Src enhances the formation and levels of HER2/HER3 heterocomplex, resulting in increased downstream signaling and biological functions (i.e., cellular motility and anchorage-independent growth) [73].

## 4. Current Status of c-Src Inhibitors and Their Effects in Drug Resistance

Although genetic mutations in SRC gene are not driver events in tumorigenesis, several studies documented its de-regulation in diverse cancer types, inducing alteration of many signaling pathways. For this reason, the development of selective c-Src inhibitors has become an attractive research topic [74].

Nowadays, five c-Src ATP competitive multikinase inhibitors are FDA-approved for their use in several cancer types or currently tested in clinical trials, most for the treatment of hematological malignancies, such as chronic myelogenous leukemia or acute lymphoblastic leukemia. A detailed summary of the main targets and clinical applications of c-Src inhibitors—bosutinib, dasatinib, ponatinib, vandetanib, and saracatinib—is reported in Table 1. In this review, we mainly describe the synergistic effect of c-Src inhibitors in combination with EGFR inhibitors and/or with chemotherapeutic drugs on solid tumors, so that we have focused our attention on dasatinib, saracatinib and bosutinib, typically tested in lung, pancreatic, colorectal and breast cancer (Table 1).

Despite the anti-tumoral effects of c-Src inhibitors reported in pre-clinical studies [75,76,77,78] the controversial outcomes of recent clinical trials highlighted the need to identify novel predictive biomarkers of tumor response to c-Src inhibitors. The high expression of Estrogen Receptor (ERα) and HER2 were firstly reported as favorable indicators for the use of c-Src inhibitors in breast cancer cell lines [75,76]. Recently, it has been reported that triple negative breast cancer (TNBC) cells also showed sensitivity to c-Src inhibitors [79]. Lou and colleagues reported that c-Src inhibitors were able to prevent TNBC invasive ability by determining a significant decrease in vimentin expression. Thus, authors suggested vimentin as a predictive biomarker to stratify breast cancer patients in clinical trials testing c-Src inhibitors [79].

Beyond its well documented effect on tumor growth and invasion, c-Src has also a crucial role in the acquisition and maintenance of resistance to many chemotherapeutic drugs, thus encouraging the concurrent use of c-Src inhibitors in combination with different cytotoxic agents. In NSCLC, it was demonstrated that vinorelbine resistant cells showed a hyper-activation of focal adhesion pathways, including SFKs and Protein Kinase B (PKB or AKT), and that the treatment with SFKs inhibitor saracatinib increased tumor cell sensitivity to vinorelbine [80]. In colorectal cancer (CRC) dasatinib treatment is able to re-sensitize cells to oxaliplatin and fluoropyrimidines, that are among the most reliable therapies for both early and late stage CRCs. Perez and colleagues showed that high levels of phosphorylated Src on Tyr419 increased resistance to oxaliplatin, but not to 5-fluorouracil. Further, dasatinib rescued this effect and the combination with oxaliplatin inhibited tumor growth, in patient derived xenografts (PDXs) from human CRC liver metastasis [81]. Similarly, the highly potent pan-SFK inhibitor A-770041 and/or short hairpin RNAs (shRNAs) against SRC mRNA reduced c-Src expression in osteosarcoma cell lines and enhanced their sensitivity to doxorubicin or paclitaxel [82].

c-Src kinase has been shown to modulate the efficacy of linsitinib, an Insulin-like Growth Factor Receptor 1 (IGF-1R) inhibitor. Min and colleagues reported that in NSCLC cells expressing high levels of pSrc, linsitinib had a slight effect on c-Src, EGFR and AKT kinase activity, followed by a rapid Src-dependent EGFR activation. In contrast, low pSrc levels were associated with higher sensitivity to linsitinib. The combined treatment of linsitinib with dasatinib successfully abrogated IGF-1R, AKT and c-Src activation and affected cell proliferation, anchorage independent colony formation and increased apoptosis in NSCLC cells and had anti-proliferative effects also in vivo [83].

## 5. The Role of c-Src in Tumor Resistance to EGFR Inhibitors

**Figure 1:**
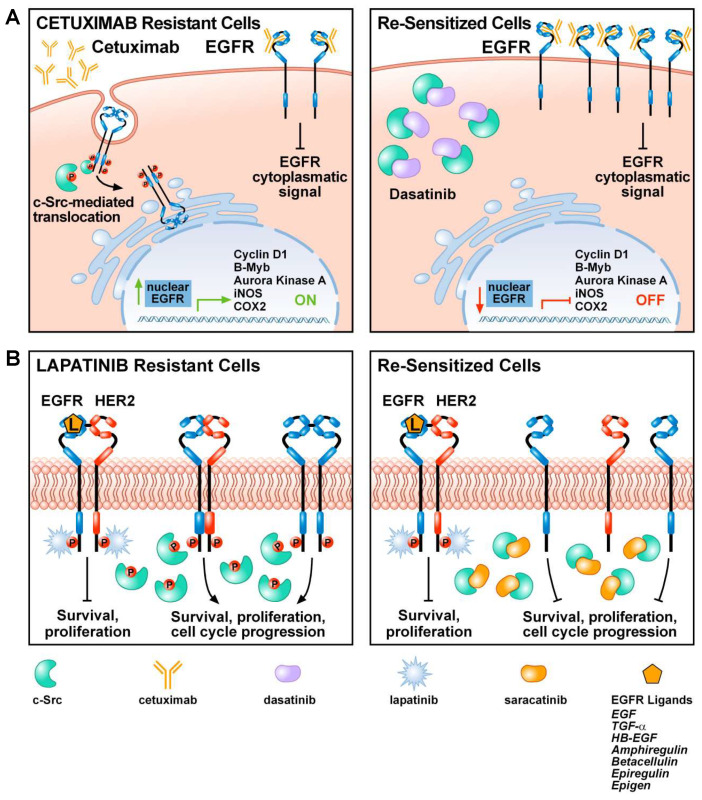
c-Src mediates mechanisms of resistance to epidermal growth factor receptor (EGFR) inhibitors: (**A**) Cetuximab resistant cells express high levels of c-Src protein. Although cetuximab might abrogate EGFR signaling from the plasma membrane, most EGFRs are translocated to the nucleus in a Src-dependent manner. Here, EGFR activates proliferative signals by modulating Cyclin D1, B-myb, Aurora kinase K, INOS, COX2 (left panel). c-Src inhibition with dasatinib reduces EGFR nuclear translocation, re-sensitizing cells to cetuximab treatment (right panel). (**B**) In lapatinib resistant breast cancer cells, c-Src over-expression induces EGFR/HER2 heterodimers activation, bypassing the lapatinib-dependent inhibition of survival and proliferation pathways. In particular, c-Src phosphorylation of EGFR Y845 boosts down-stream signaling pathways to EGFR homo and heterodimers activation (left panel). Cell treatment with c-Src inhibitor saracatinib restores lapatinib sensitivity and overcome EGFR and its related oncogenic pathways activation (right panel).

The introduction of anti-EGFR antibodies and EGFR tyrosine kinase inhibitors (TKIs) in clinical practice drastically improved the prognosis of patients affected by various cancer types, especially lung cancer, the first leading cause of cancer death worldwide. Nevertheless, primary or acquired resistance to EGFR inhibitors is the most common cause of cancer relapse and progression. Therefore, the identification of novel therapeutic targets is an unmet clinical need. Beyond the well-established role of Src kinases family in tumor initiation, progression and invasion, c-Src activation plays a key role also in the acquisition and maintenance of resistance to EGFR inhibitors in human lung, breast, colorectal and pancreatic cancer [84,85,86,87]. It has been proposed that c-Src kinase mediates the nuclear translocation of EGFR, an event that contributes to the acquired resistance to the anti-EGFR antibody cetuximab in colorectal cancer and head and neck squamous cell carcinoma (HNSCC; Figure 1). Treatment of lung cancer cell lines resistant to the EGFR monoclonal antibody cetuximab with the c-Src inhibitor dasatinib, led to a loss of nuclear EGFR and a re-sensitization to cetuximab treatment (Figure 1) [88]. NSCLC cell lines, harboring activating mutation in EGFR exon 19 (E746-A750), with acquired resistance to afatinib, a second-generation EGFR-TKI, become cross-resistant also to first-generation EGFR-TKIs, gefitinib and erlotinib, and to third-generation EGFR-TKI, osimertinib. Interestingly, EGFR-TKIs resistant cells upon treatment with dasatinib or transfection with small interfering RNAs (siRNAs) targeting SRC mRNA, significantly decreased AKT activation, cell survival and migration, indicating that Src inhibitors might overcome resistance to EGFR inhibitors in lung cancer cells [89].

Both saracatinib and dasatinib have been proposed as therapeutic agents for NSCLC. However, disappointing results from Phase I/II clinical trials have emerged, likely due to the enrolment of molecularly unselected patients with advanced NSCLC. We previously demonstrated that different c-Src inhibitors act through several mechanisms in sensitive or erlotinib-resistant NSCLC models and that the combination of c-Src or MEK inhibitors with EGFR-TKI (in EGFR or RAS mutated models, respectively) leads to higher benefits compared to single treatment in NSCLCs. Furthermore, since a few therapeutic options are available for EGFR wt/Ras-mut NSCLC, the combination of dasatinib plus MEK inhibitor selumetinib may be a valuable strategy in the clinical setting [90]. In addition, ~50% of NSCLC patients developing resistance to EGFR-TKIs shows a T790M secondary point mutation in the EGFR gene. Exploring differences in tyrosine-phosphorylation profiles—by immunoaffinity purification of tyrosine-phosphorylated peptides followed by mass spectrometry—between TKI-sensitive and gefitinib resistant (GR) NSCLCs harboring T790M, Yoshida and colleagues identified MET, Insulin-like Growth Factor (IGF), AXL and c-Src kinase as possible mediators of resistance to gefitinib. Authors assessed that neither erlotinib or afatinib could affect c-Src phosphorylation. In contrast, the combination of dasatinib with afatinib abrogated c-Src phosphorylation and downstream phosphorylation of AKT and ERK. More interestingly, the combination of dasatinib with afatinib or T790M-selective EGFR-TKI (WZ4006) significantly reduced proliferation in NSCLC cell lines. In conclusion, authors asserted the potential efficiency of combination therapy of dasatinib and afatinib in NSCLC patients with acquired EGFR-TKI resistance associated with T790M [91].

As for lung, breast and colorectal cancer, even in oral squamous cell carcinoma (OSCC) EGFR activation plays an important role in cancer progression and correlates with poor prognosis. In OSCC cell lines differently sensitive to EGFR-TKIs, resistance to the EGFR inhibitor cetuximab is related to HER3 activation. Since c-Src inhibitor-1 (a selective dual site Src tyrosine kinase inhibitor of c-Src and Lck) was able to affect HER3 phosphorylation, they demonstrated that the combination of cetuximab with c-Src inhibitor-1 led to a concurrent decrease of EGFR and HER3 phosphorylation, significantly reducing tumor cell growth [92]. c-Src inhibitor-1 efficacy in overcoming resistance to EGFR inhibitors was also reported in HER2+ gastric and biliary tract tumors: cancer cells resistant to the anti-HER2 antibody trastuzumab showed higher c-Src and FAK phosphorylation levels, compared to sensitive cells. Interestingly, the concomitant treatment of resistant cells with trastuzumab and bosutinib impaired cell growth, migration and cell-cycle progression, revealing c-Src contribution to such resistance [93].

Furthermore, in HER2+ breast cancer cells, the combination of lapatinib and saracatinib significantly affected cell proliferation, survival, motility, migration, and invasion [84]. Interestingly, it has been demonstrated that the combination of HER2 and c-Src inhibitors drastically reduced lung metastases in nude mice injected with lapatinib-resistant breast cancer cells. Moreover, lapatinib-resistant cells showed increased c-Src expression levels, which in turn activate EGFR on Y845 with a molecular mechanism described above (Figure 1). In lapatinib-resistant cells the use of a single-agent drug did not exert any relevant biologic effect, but the combination of saracatinib and cetuximab inhibited proliferation, migration, and invasion, due to the interference in Src-mediated EGFR activation (Figure 1). These findings have relevant impact on clinical settings since the co-expression of HER-2 and EGFR is observed in ~10% to 36% of breast cancer patients and it is generally associated with a poorer prognosis. Moreover, the survival of breast cancer patients with high phosphorylated HER2 or both HER2 and EGFR proteins is significantly shortened [94].

In conclusion, these pre-clinical data show not only the key role of Src kinase family in the acquisition and maintenance of tumor resistance to EGFR inhibitors, especially in lung cancer, but also help drive which approaches to prioritize for novel clinical therapeutic combinations aimed to overcome the effects of such resistance.

## 6. Combined Therapy of c-Src and EGFR Inhibitors in Recent Clinical Trials

The encouraging preclinical data pushed forward testing the combination of EGFR and c-Src inhibitors in clinical trials, particularly in cancer patients who progressed after EGFR inhibitors. In 2019, Creelan and colleagues conducted a Phase I clinical trial testing the combination of dasatinib and afatinib on 25 lung cancer patients with activating mutations on EGFR exon 19 or 21 or disease progression following prior EGFR-TKI therapy (Table 2; [95]). Despite the good tolerability, dasatinib-based therapy showed no significant benefits in this cohort of patients, likely due to the short drug half-life in plasma. Moreover, also dasatinib and afatinib combination led to less or no positive clinical responses (Table 2; [95]). Currently, other two clinical trials are ongoing in NSCLC patients, (Table 3; NCT00444015 and NCT02954523). The first one is a single site dose escalation trial of erlotinib with dasatinib on 34 patients with previously treated advanced stage (Stage IIIB/IV disease) NSCLC aimed to determine the safety, tolerability and the maximum tolerated dose (MTD) of erlotinib in combination with dasatinib (NCT00444015). The second one is a Phase I/II trial that will evaluate the effects of the third generation EGFR-TKI, osimertinib in combination with dasatinib in EGFR mutant NSCLC patients who developed resistance to the first-generation EGFR-TKIs, erlotinib and gefitinib, aiming to assess serum biomarkers to monitor clinical outputs upon c-Src inhibitor treatments. Since authors previously demonstrated that c-Src activation led to Cripto-1 over-expression in EGFR mutant NSCLC, contributing to the intrinsic resistance to EGFR-TKIs, the ongoing trial aims to define the MTD of dasatinib, administered in combination with osimertinib, and to assess the role of Cripto-1 protein as predictive biomarkers of response (Table 3; NCT02954523). The combination of dasatinib with EGFR inhibitors is under investigation in other cancer types, as the ongoing Phase I clinical trial NCT00996723 evaluating the combination of the vandetanib and dasatinib during and after radiation therapy in diagnosed diffuse intrinsic pontine glioma (Table 3).

Interesting results have been reported in a Phase I clinical trial in pancreatic adenocarcinoma. Between July 2012 and October 2015, 19 patients with metastatic or locally advanced pancreatic adenocarcinoma, not previously treated with gemcitabine, were eligible for the enrollment in order to investigate the MDT of dasatinib used in combination with fixed doses of erlotinib and gemcitabine. The triplet resulted in stable disease (SD) in 69% of patients, while 8 out of 13 patients (62%) showed reduction in tumor size. The detailed results accomplished with this trial—summarized in Table 2—showed no relevant side effects for the combination of gemcitabine with erlotinib and dasatinib and exhibited encouraging preliminary results in advanced pancreatic adenocarcinoma (Table 2; [96]).

On the basis of pre-clinical data suggesting the synergistic effects of dasatinib with oxaliplatin and cetuximab in advanced metastatic colorectal cancer, a Phase Ib/II clinical trial was conducted to test the effect of the oral c-Src inhibitor, dasatinib, in combination with the chemotherapeutic regimen FOLFOX and the anti-EGFR cetuximab. As described in Table 2, 30 patients affected by colorectal adenocarcinoma, with metastatic diseases and different KRAS mutational statuses, were enrolled in the Phase Ib portion of the study. The enrolled population of patients was previously heavily treated, with an average of four previous lines of therapy. Overall, 90% of patients were previously treated with oxaliplatin and 80% of them were also exposed to anti-EGFR treatment. The primary goals of Phase Ib were to determine the MTD and dose-limiting toxicity of dasatinib, cetuximab and FOLFOX6 combination and their effects on the biological activity of c-Src. The treatment regimen consisted in standard doses of cetuximab, standard FOLFOX6 regimen and increasing doses of oral dasatinib in different cohorts: 100, 150, 200 mg/day administered continuously. As the dasatinib dose for further studies, 150 mg/day was chosen, although patients were not able to tolerate it for more than 3 months, because of myelosuppression and fatigue. Unexpectedly, the pharmacodynamics studies showed that this treatment regimen did not affect c-Scr activity, probably due to an oxaliplatin-dependent increase in p-Src levels that dasatinib was not able to rescue. In the Phase II part of the study, patient response and disease progression were screened based on KRAS mutational state. In the KRAS wild-type cohort, overall response rate (ORR) of 30% was reported; no significant responses were recorded in the KRAS mutated cohort of mCRC (metastatic colorectal cancer) patients. In conclusion, this study showed that the combination of FOLFOX with cetuximab and dasatinib in heavily pre-treated mCRC patients brings only modest benefits, probably due to the inability of dasatinib to completely abrogate c-Src phosphorylation (Table 2; [97]). HNSCC has no predictive biomarkers described so far, even if EGFR targeting still remains a valid therapeutic option. Preclinical models of HNSCC described c-Src kinase involvement in tumor resistance to EGFR inhibitors [100,101]. These data were supported by the finding that high phosphorylation levels of c-Src in HNSCC patients represent predictive marker of poor response to erlotinib [102]. Based on these results, a randomized, placebo-controlled window trial on the use of c-Src inhibitor dasatinib in combination with erlotinib was conducted to assess dasatinib capability to overcome tumor resistance to erlotinib in HNSCC patients. The study proved that even if erlotinib significantly decreased tumor size in tumor patients, the combined therapy of dasatinib with erlotinib had no additive or synergistic clinical benefits. Authors observed that, during dasatinib treatment, tumor progression was associated with a hyper-activation of STAT3, an event that could be considered as a de novo acquired mechanism of resistance to dasatinib in HNSCC patients [103]. Based on the emerging role of IL6/JACK/STAT3 pathway in dasatinib-resistant HNSCC patients, a phase II clinical trial of combined therapy of cetuximab plus dasatinib in metastatic HNSCC was conducted, measuring pre- and post-treatment patients’ serum levels of Interleukin-6 (IL-6) [98]. Interestingly, authors found a significant clinical improvement, in terms of increased overall survival and better response (Table 2), in patients with low IL-6 serum levels so that it could be considered a clinical predictive biomarker for response to cetuximab and dasatinib combined therapy in HNSCC patients.

Finally, based on the above described encouraging pre-clinical data of c-Src kinase and HER2 receptor co-targeting in breast cancer, Ocana and collaborators conducted a Phase II clinical trial combining dasatinib with the anti-HER2 trastuzumab and paclitaxel (Table 2). Associated with promising clinical outcomes, pharmacodynamic analysis of tumor tissues, blood samples and skin biopsies of 29 patients enrolled, revealed a significant decrease of phospho-Src, phospho-ERK and phospho-AKT in epidermal keratinocytes. These results definitely encouraged dasatinib use in combination with trastuzumab and paclitaxel, due to good toxicity profile and a high efficacy, as revealed by an objective response rate of 79.3% [99], however, because of the absence of a control arm, further investigations in subsequent Phase II/III controlled trials are required.

## 7. Conclusions

The development and the use of three generations of EGFR inhibitors in the clinical setting significantly improved the prognosis of NSCLC cancer patients, in particular in ~10% to 15% of cases of white patients and even 50% of cases of Asian NSCLC patient, harboring activating EGFR mutations [104]. Unfortunately, the presence of innate or the onset of acquired resistance to EGFR inhibitors remains the most common cause for cancer relapse and mortality, highlighting the importance to investigate the complex network of cancer resistance mechanisms. The evidence that c-Src over-expression is reported in ~50 to 80% of NSCLC patients led to increased research efforts for investigating the effects of concurrent c-Src/EGFR inhibition on cancer growth and dissemination. Several pre-clinical data, here reported, showed encouraging results, corroborating c-Src inhibitors capability to overcome tumor resistance to EGFR-inhibitors in different cancer types. However, the last Phase I/II clinical trials combining c-Src and EGFR inhibitors provided controversial outcomes, as summarized in Table 2. In lung, mCRC and HNSCC cancer patients, despite the good tolerability, combination therapy of dasatinib with EGFR inhibitors leads to no consistent clinical responses. However, encouraging outcomes emerged from breast and advanced pancreatic patients where high response rates were observed. Several authors assert that the lack of clinical benefits of combined therapies depends mainly on two major points: i) an incomplete abrogation of c-Src hyper-activation and ii) the enrolment of molecular un-characterized patients. Clinical trials, in both mCRC and HNSCC, clearly showed that the ORR of the combined therapy increased in KRAS wt or in patients with low serum levels of IL-6, respectively. In this context, the importance of further molecular investigation is also highlighted in a current ongoing clinical trial enrolling lung cancer patients, which is evaluating the expression of Cripto-1 following the treatment with dasatinib and osimertinib.

In conclusion, recent research efforts clearly showed the need to identify specific subsets of patients, resistant to EGFR inhibitors, using predictive biomarkers of response in order to strongly enhance the clinical outcomes from the combined therapy of c-Src/EGFR inhibitors.

## Figures and Tables

**Table 1:** c-Src ATP competitive inhibitors.

Drugs	Molecular Targets	Clinical Applications
**Bosutinib**	BCR-Abl, c-Src, Lyn, Hck, Kit, PDGFR	CML, ALL + clinical trials for breast cancer, glioblastoma
**Dasatinib**	BCR-Abl, SFKs, Arg, c-KIT, EGFR, PDGFR, DDR1, DDR2, c-FMS, ephrin receptors, TEK, BTK, EphA2	CML + clinical trials for ALL, breast, colorectal, endometrial, head and neck, ovarian, and small cell lung cancers, glioblastoma, melanoma, and NSCLC
**Ponatinib**	BCR-Abl, SFKs, VEGFR, PDGFR, FGFR, Eph, Kit, RET, Tie2, Flt3	CML, ALL + clinical trials for endometrial, GIST, hepatic biliary, small cell lung, and thyroid cancers
**Vandetanib**	RET, SFKs, EGFR, VEGFRs, Brk, Tie2, EphR	medullary thyroid carcinoma
**Saracatinib (AZD0530)**	c-Src, BCR-Abl	Clinical trial for SCLC, NSCLC, colorectal, gastric, ovarian and metastatic osteosarcoma

CML = chronic myelogenous leukemia; ALL = acute lymphoblastic leukemia; GIST = gastrointestinal stromal tumor; SCLC = small cell lung cancer; NSCLC = non-small cell lung cancer.

**Table 2:** Clinical trials combining EGFR inhibitors and c-Src kinase inhibitors.

Authors	Disease	Clinical Trial/Phase	Drugs	Total No. of Patients	Mutational State/Drug Resistance	Results
Creelan BC et al. [95]	Lung Cancer, Non-small cell lung cancer (NSCLC)	NCT01999985 Phase IA, IB	saracatinib, afatinib	25	EGFR mut or EGFR TKI resistant	mPFS = 3.7 months (95% CI, 2.3–5.0) mOS = 14.7 months (95% CI, 8.5–20.9)
Cardin DB et al. [96]	MPAC, RPA, Stage III and IV Pancreatic Cancer	NCT01660971 Phase I	dasatinib, erlotinib, gengitabine	19	NA	DCR = 69%, mPFS = 3.6 months (95% CI, 3.8 to NA), OS = 8 months (95% CI, 4.4 to 17)
Parseghian CM et al. [97]	Metastatic Colon-Rectal Cancer	NCT00501410 Phase IB/II	dasatinib, FOLFOX	77	KRAS c12/13^mut^ KRAS c12/13^wt^	ORR = 30% (95% CI, 0.17 to 0.45) only KRASwt; stable disease 23%; (95% CI, 0.12 to 0.38) mOS = 6.7 months in all patients
Stabile LP et al. [98]	Recurrant/metastatic HNSCC	NCT01488318 Phase II	dasatinib, cetuximab	21	Progression after cetuximab	SD= 36%, PD = 57%; mPFS = 1.7months (90%CI, 1.4–3.9 months); mOS = 5.1 months (90% CI, 4.2–11.5 months)
Ocana A et al. [99]	Metastatic Breast Cancer	NCT01306942 Phase I/II	dasatinib, paclitaxel, trastuzumab	39	HER2 +	ORR = 79.3% (*n* = 23; 95% CI 60.3 to 92.0); CBR = 82.8% (*n* = 24; 95% CI 64.2–94.2); mPFS = 23.9 months (95% CI 10.3–NR); TTP = 23.9 months (95% CI 14.9-NR): RD = NR

CI = Confidential interval; HNSCC = head and neck squamous cell carcinoma; mCRC = metastatic colorectal cancer; mPFS = median progression free survival; MPAC = metastatic Pancreatic Adenocarcinoma; NR = not reached; OL = open label; ORR = overall response rate; OS = overall survival; PR = partial response; R = randomized; RD= Response Duration; RPA = recurrent pancreatic carcinoma; TTP = time to progression; NA = Not Available

**Table 3:** Ongoing Clinical Trials.

Clinical Trial ID	Disease Condition	Study Phase	Combination Drugs	Status at Time of Search
NCT00444015	Stage IIIB/IV disease Recurrent NSCLC	Phase I	Erlotinib + Dasatinib	Completed
NCT02954523	EGFR mut NSCLC	Phase I/II	Dasatinib + osimertinib	Active, not recruiting
NCT00996723	Diffuse Intrinsic Pontine Glioma	Phase I	Vandetanib + dasatinib	Completed
